# Body Mass Index, Waist Circumference, Readmission, and Healthcare Costs After Influenza- or Pneumonia-Related Hospitalization: A Sex-Stratified Nationwide Cohort Study in Korea

**DOI:** 10.3390/healthcare14162529

**Published:** 2026-08-13

**Authors:** Kangho Suh, Sujin Lee, Seung-Mi Lee

**Affiliations:** 1 School of Pharmacy, University of Pittsburgh, Pittsburgh, PA 15261, USA; kas551@pitt.edu; 2 College of Pharmacy, Daegu Catholic University, Gyeongsan 38430, Republic of Korea; sujinlee20@cu.ac.kr

**Keywords:** obesity, body mass index, waist circumference, influenza, pneumonia, readmission, healthcare costs, Korea

## Abstract

**Highlights:**

**What are the main findings?**

- Body mass index and waist circumference showed different category-specific associations with post-discharge outcomes in sex-stratified analyses.
- Higher waist circumference categories were associated with greater all-cause readmission burden in the female stratum, whereas the corresponding patterns were less consistent for other outcomes.

**What are the implications of the main findings?**

- The findings do not establish that waist circumference provides independent or incremental predictive information beyond body mass index.
- Further studies using mutually adjusted models, formal interaction tests, competing-risk methods, and prediction analyses are required before clinical application.

**Abstract:**

**Background/Objectives:** Obesity is associated with respiratory infection risk, but its relationship with post-discharge healthcare burden remains unclear. This study examined the associations of body mass index (BMI) and waist circumference (WC), evaluated separately, with hospitalization and post-discharge outcomes in analyses stratified by sex among South Korean adults with claims-defined hospitalizations involving influenza or pneumonia. **Methods:** This retrospective cohort study used the 2018 National Health Insurance Service (NHIS) Customized Research Database linked to health-screening data. Adults aged 20–89 years with claims-defined hospitalizations involving influenza or pneumonia were included if BMI and WC measurements were available from the most recent NHIS health-screening examination conducted within two years before the index admission. Length of stay and index hospitalization costs were summarized descriptively. Cox proportional hazards models were used to estimate hazard ratios (HRs) for all-cause and pneumonia-related readmission within two years after discharge, and two-part models were used to estimate readmission-related costs, expressed in US dollars (USD). **Results:** The cohort included 175,774 patients, of whom 80,257 (45.7%) were male. Category-specific associations varied according to the anthropometric measure, outcome, and sex stratum. In female patients, the highest WC category had the largest estimated all-cause readmission HR (HR 1.195, 95% confidence interval 1.157–1.235), and the highest model-based mean cost of the first all-cause readmission was also observed in this category (USD 1407.4). In male patients, several higher BMI categories had lower estimated readmission HRs than the reference category; however, these estimates should not be interpreted as protective effects because competing mortality was not accounted for using competing-risk methods. Patterns for pneumonia-related readmission and costs were less consistent. **Conclusions:** BMI and WC showed category-specific associations with readmission, while model-based mean readmission costs varied across anthropometric categories and sex strata. Higher WC categories were associated with higher all-cause readmission HRs in the female stratum; however, the independent or incremental prognostic value of BMI and WC was not evaluated.

## 1. Introduction

Obesity has become one of the most pressing global public health challenges, with its prevalence steadily increasing worldwide. In 2020, an estimated 2.2 billion adults, accounting for 42% of the global adult population, were living with overweight or obesity. This number is projected to increase to 3.3 billion, or over 54% of adults, by 2035 [1]. In Korea, the prevalence of obesity has continued to rise and is expected to further increase, with important implications for chronic disease burden and healthcare utilization [2].

In addition to its well-established role in metabolic and cardiovascular diseases, obesity has emerged as an important risk factor for infectious diseases, including respiratory tract infections. Excess adiposity can impair host immune defense through mechanisms such as chronic low-grade inflammation, altered cytokine profiles, impaired lung mechanics, and dysregulated immune cell function [3,4]. These effects are particularly relevant in influenza and pneumonia, which remain major causes of morbidity, mortality, hospitalization, and healthcare use worldwide [5].

Recent studies suggest that obesity increases susceptibility to respiratory infections and may be associated with worse clinical outcomes, including more severe illness and greater healthcare utilization [6,7,8]. Some studies have reported inverse associations between BMI and mortality among hospitalized patients with pneumonia. However, these findings may be influenced by frailty, illness-related weight loss, selection processes, and competing mortality and should not necessarily be interpreted as protective effects of obesity [9]. This heterogeneity indicates that obesity-related risk may differ by population, outcome, and the anthropometric measure used.

The relationship between adiposity and infection outcomes may also vary by sex and by the distribution of adiposity. Sex-related differences in fat distribution, adipose tissue biology, immune responses, and healthcare-seeking behavior provide a rationale for presenting the associations separately by sex [10,11,12]. Body mass index (BMI) reflects general adiposity but does not distinguish lean mass from fat mass or indicate central fat distribution. BMI and waist circumference (WC) reflect different aspects of adiposity. In the present study, they were evaluated separately, without assuming that either measure provides independent or incremental information beyond the other [4,13,14].

Influenza and pneumonia were grouped pragmatically to define a claims-defined cohort of hospitalizations involving acute respiratory infection and systemic antibacterial treatment. This grouping was not intended to imply etiological, clinical, or prognostic equivalence between the two conditions. Nevertheless, because pneumonia represents a clinically important and more specific respiratory infection outcome, pneumonia-related readmission was evaluated separately from all-cause readmission.

In South Korea, the National Health Insurance Service (NHIS) provides comprehensive claims data covering nearly the entire population, and its linkage with national health-screening data enables assessment of pre-admission anthropometric measures [15,16,17]. This data environment provides an opportunity to evaluate real-world associations of BMI and WC with length of stay, readmission, and healthcare costs after hospitalization for influenza or pneumonia.

This study aimed to examine the associations of BMI and WC, evaluated in separate models, with hospitalization and post-discharge outcomes in analyses stratified by sex among South Korean adults with claims-defined hospitalizations involving influenza or pneumonia. Specifically, length of stay, index hospitalization costs, all-cause and pneumonia-related readmission within two years after discharge, and readmission-related healthcare expenditures were assessed. The study was intended to describe category-specific epidemiological associations and generate hypotheses for subsequent analyses incorporating competing risks, mutually adjusted anthropometric measures, and formal prediction methods.

## 2. Materials and Methods

### 2.1. Study Design and Data Source

This retrospective cohort study used data from the NHIS Customized Research Database in South Korea. The NHIS provides comprehensive healthcare coverage for nearly the entire Korean population and maintains administrative claims data, including demographic characteristics, diagnostic codes, treatment procedures, prescription records, medical institution type, and results from routine national health-screening programs [15,17]. Health-screening records that provided BMI and WC measurements were used to classify general and central obesity.

The cohort year 2018 was selected to allow for complete two-year follow-up before major changes in respiratory infection diagnosis, admission, and healthcare utilization patterns related to the COVID-19 pandemic.

### 2.2. Study Population and Index Hospitalization

The cohort consisted of adults aged 20–89 years with claims-defined hospitalizations involving influenza or pneumonia in 2018. Eligible hospitalizations were identified when an influenza (International Classification of Diseases, 10th Revision [ICD-10] codes J09-J11) or pneumonia (ICD-10 codes J12-J18) code was recorded in one of the eligible diagnosis positions at admission. Systemic antibacterial treatment classified under the World Health Organization Anatomical Therapeutic Chemical (ATC) J01 was required as an operational inclusion criterion based on claims data. This criterion was intended to improve specificity but did not confirm bacterial pneumonia or establish that respiratory infection was the principal reason for admission. In claims data, J01 exposure was identified using drug codes mapped to ATC classification codes.

Individuals with clinical conditions that could confound the association between obesity and infection outcomes, including malignant neoplasms, organ transplantation history, hemophilia, or pregnancy- and childbirth-related hospitalization, were excluded. Patients without valid BMI and WC measurements from NHIS health screenings conducted within two years before the index hospitalization were also excluded.

For patients with multiple eligible hospitalizations in 2018, the first eligible hospitalization was defined as the index hospitalization.

### 2.3. Exposure Assessment: Body Mass Index and Waist Circumference

Obesity was evaluated using BMI and WC obtained from NHIS general health-screening records conducted within two years before the index hospitalization. The NHIS general health-screening program is routinely provided at approximately two-year intervals. Accordingly, BMI and WC measurements obtained during the two years before the index hospitalization were considered eligible. Only examinations performed before the index admission were included. When more than one eligible examination was available, BMI and WC from the examination date closest to the index hospitalization were selected. Thus, the two-year period represented the maximum look-back window, and the most recent available pre-admission measurements were used. BMI was calculated as weight in kilograms divided by height in meters squared (kg/m^2^) and categorized using criteria proposed by the World Health Organization for the Asia-Pacific region and Korean Society for the Study of Obesity (KSSO) clinical guidelines [18,19]. Participants were classified into six BMI groups: underweight (<18.5 kg/m^2^), normal weight (18.5–22.9 kg/m^2^), overweight (23.0–24.9 kg/m^2^), obesity class I (25.0–29.9 kg/m^2^), obesity class II (30.0–34.9 kg/m^2^), and obesity class III (≥35.0 kg/m^2^).

WC was used as a proxy for abdominal obesity and categorized using sex-specific KSSO thresholds [13]. Male participants were classified as Category 1 (<80 cm), Category 2 (80.0–84.9 cm), Category 3 (85.0–89.9 cm), Category 4 (90.0–94.9 cm), Category 5 (95.0–99.9 cm), and Category 6 (≥100 cm). Female participants were classified as Category 1 (<75 cm), Category 2 (75.0–79.9 cm), Category 3 (80.0–84.9 cm), Category 4 (85.0–89.9 cm), Category 5 (90.0–94.9 cm), and Category 6 (≥95 cm). Because measurements were obtained before hospitalization, exposure misclassification may have occurred if weight or WC changed between the screening date and admission.

### 2.4. Covariates

Baseline characteristics were extracted from claims and health-screening records and included age, sex, BMI, WC, income level (quartiles), residential area (metropolitan or non-metropolitan), Charlson Comorbidity Index (CCI), and type of medical institution (clinic, hospital, general hospital, or tertiary hospital) [20]. These variables were used to describe the study population. Age, Charlson Comorbidity Index score, income level, and residential area were included as covariates in the regression models.

### 2.5. Clinical and Economic Outcomes

Clinical outcomes included length of stay, index hospitalization costs, and readmission events within two years after discharge. Length of stay was defined as the number of days from admission to discharge for the index hospitalization and was summarized descriptively by BMI and WC category. Index hospitalization costs represented direct medical expenditures incurred during the index admission, calculated from NHIS claims data.

Costs recorded in Korean won (KRW) were converted to US dollars (USD) using the 2018 annual average exchange rate of KRW 1100.19 per USD. Therefore, costs are presented as 2018 USD values unless otherwise stated.

All-cause readmission was operationally defined as the first subsequent inpatient hospitalization recorded after the index discharge date. Pneumonia-related readmission was defined as the first subsequent claims-defined inpatient hospitalization with a primary or secondary pneumonia diagnosis (ICD-10 codes J12-J18) during follow-up. No minimum post-discharge grace period was applied, and planned and unplanned admissions were not distinguished. Consecutive claims were consolidated into a single hospitalization episode; however, interhospital transfers were not consolidated across institutions. Readmission-related cost was defined as the medical expenditure derived from claims data for the first qualifying readmission occurring within two years after discharge. Pneumonia-related readmission cost was defined as the medical expenditure associated with the first qualifying pneumonia-related readmission within the two-year follow-up period.

### 2.6. Statistical Analysis

Descriptive statistics were used to summarize baseline characteristics by sex and obesity category. Continuous variables were reported as means with standard deviations (SDs), and categorical variables were summarized using frequencies and percentages. The use of means and SDs was descriptive and did not imply normality. Group comparisons were conducted using *t*-tests for continuous variables and chi-square tests for categorical variables.

Associations between obesity-related measures and readmission were evaluated using Cox proportional hazards regression models. The models evaluated time to the first qualifying subsequent hospitalization, and death was treated as a censoring event when no earlier qualifying readmission occurred. Because death precluded subsequent readmission, the conventional Cox estimates were interpreted as cause-specific associations rather than cumulative readmission risks. Tied event times were handled using the Breslow method. The proportional hazards assumption for BMI and WC categories was assessed by visual inspection of the corresponding sex-stratified survival curves. No marked curve crossing was observed; however, no formal residual-based diagnostic was conducted. Models estimated hazard ratios (HRs) and 95% confidence intervals (CIs) for all-cause and pneumonia-related readmission. Models were adjusted for age, CCI score, income level, and residential area.

Readmission-related costs were analyzed using a two-part modeling approach to account for skewed cost distributions and zero-cost observations [21]. In the first part, logistic regression estimated the probability of incurring any readmission cost. In the second part, a generalized linear model with a gamma distribution and log-link function estimated mean costs among patients who were readmitted. For each cost outcome, 1000 resamples of the precomputed individual predicted costs were generated using unrestricted random sampling with replacement. The SD represents the standard deviation of the resampled mean estimates, whereas the reported 95% CI is the confidence interval for the mean of the 1000 resampled estimates. These intervals describe the numerical stability of the resampling procedure and should not be interpreted as percentile bootstrap confidence intervals for the full two-part model. Statistical analyses were performed using SAS version 9.4 (SAS Institute Inc., Cary, NC, USA).

## 3. Results

A total of 175,774 adult patients with claims-defined hospitalizations involving influenza or pneumonia were included, comprising 80,257 males (45.7%) and 95,517 females (54.3%). The mean age was slightly higher in males than in females (63.3 ± 16.2 vs. 61.9 ± 16.6 years, *p* < 0.001). Females had a slightly higher mean BMI (23.9 ± 3.9 kg/m^2^ vs. 23.7 ± 3.6 kg/m^2^), whereas males had a higher mean WC (84.7 ± 9.4 cm vs. 79.8 ± 10.1 cm). Pneumonia was identified in 65.6% of patients. Male patients had a slightly higher comorbidity burden and were more often admitted to tertiary hospitals, whereas female patients were more often treated in hospitals or clinics (Table 1).

**Figure 1:**
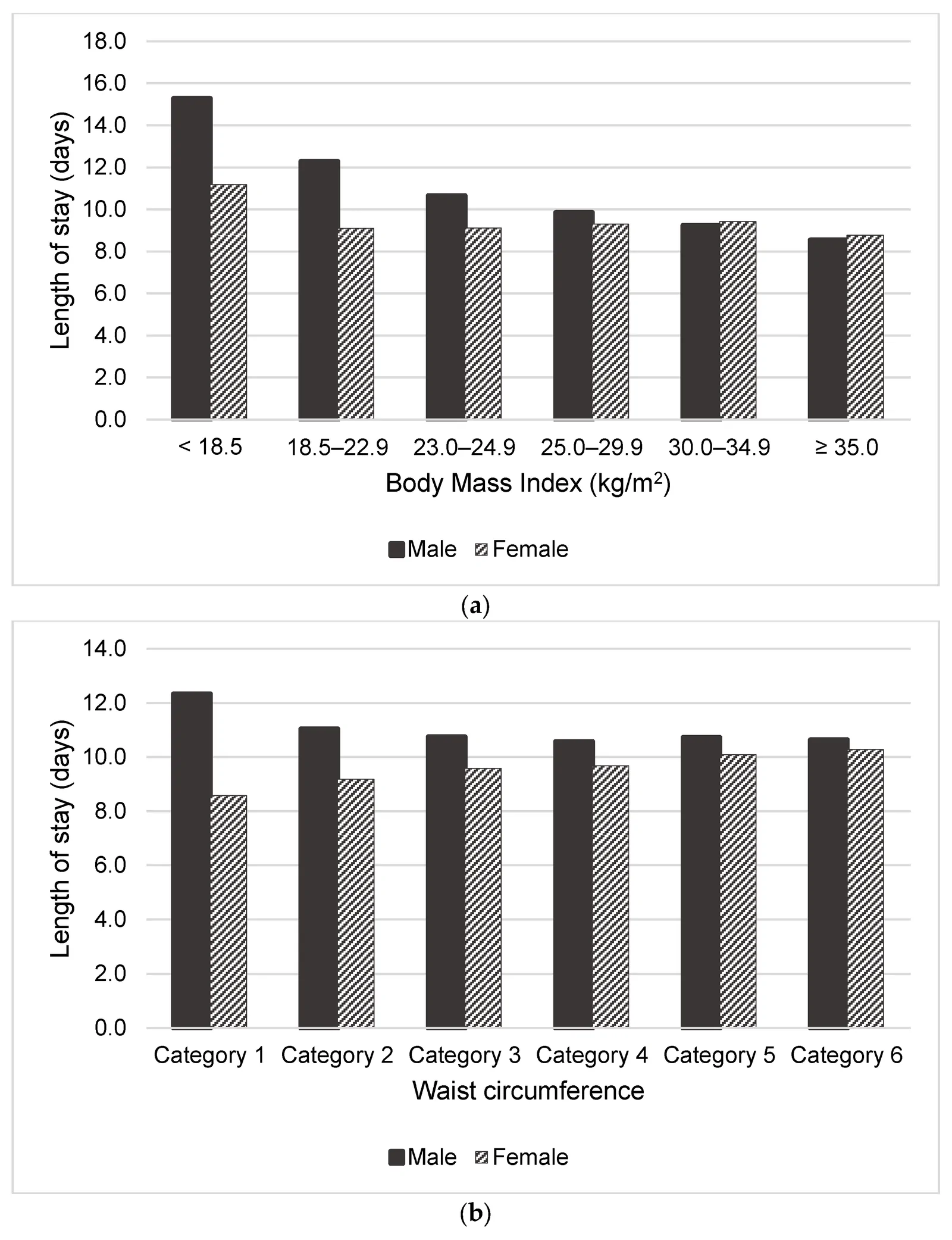
Length of stay by obesity level among adults hospitalized for influenza or pneumonia: (**a**) body mass index; (**b**) waist circumference.

Observed mean length of stay and index hospitalization costs varied descriptively across BMI and WC categories and sex strata. Among male patients, the observed mean length of stay was longest in the underweight BMI category and shorter in several higher BMI categories. A similar pattern was observed among female patients, with the mean length of stay longest in the underweight BMI category (11.2 days), relatively stable across the normal-weight, overweight, obesity class I, and obesity class II categories (9.1–9.4 days), and shortest in obesity class III (8.8 days). Across WC categories, the observed mean length of stay was generally longer in the upper categories among female patients, whereas the corresponding pattern was less pronounced among male patients (Figure 1).

**Figure 2:**
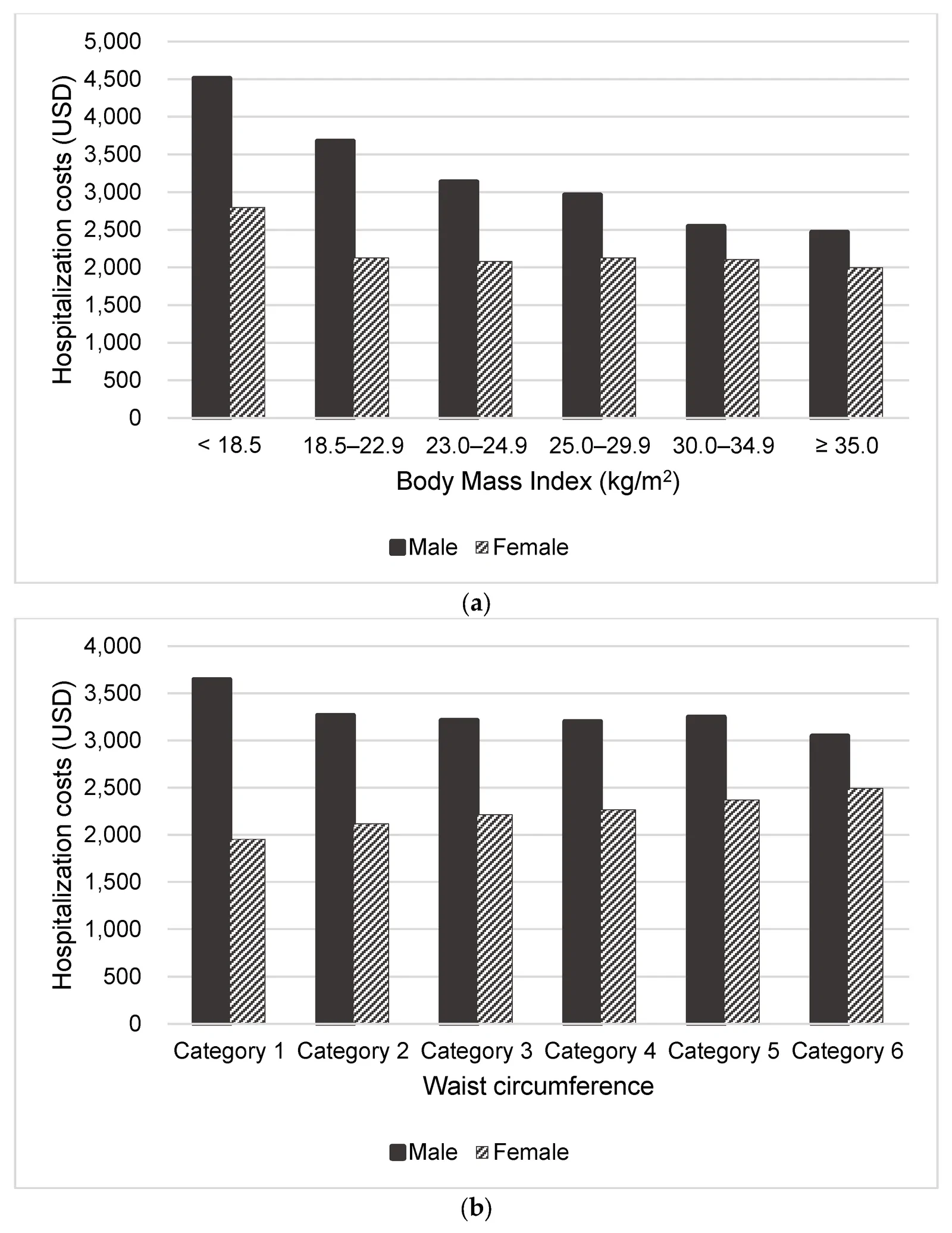
Index hospitalization costs by obesity level among adults hospitalized for influenza or pneumonia: (**a**) body mass index; (**b**) waist circumference. Costs are presented as 2018 USD.

Observed index hospitalization costs varied descriptively across BMI and WC categories and sex strata. No adjusted or inferential comparisons were performed. In males, lower mean costs were observed in several higher BMI categories, from USD 4510 in the underweight group to USD 2462 in the BMI ≥ 35.0 kg/m^2^ group. In females, costs were highest in the underweight group and generally lower across higher BMI categories. By WC, male costs were highest in Category 1 and lowest in Category 6, whereas female costs increased from USD 1953 in Category 1 to USD 2492 in Category 6 (Figure 2).

Category-specific estimates for all-cause readmission varied across anthropometric measures and sex strata (Table 2). Overall, 99,898 patients (56.8%) experienced at least one all-cause readmission, and 26,819 (15.3%) experienced at least one pneumonia-related readmission. During the two-year follow-up, 12,996 patients (7.4%) died, including 8412 of 80,257 male patients (10.5%) and 4584 of 95,517 female patients (4.8%). Among male patients, the underweight category had a higher all-cause readmission HR than the normal-weight BMI category (HR 1.091, 95% CI 1.052–1.132), whereas the overweight and obesity class I categories had lower HRs. Among male patients, WC Categories 2–4 had HRs similar to or lower than that of Category 1, whereas Category 6 had a higher HR (HR 1.072, 95% CI 1.028–1.118). In the female stratum, several higher BMI and WC categories had higher HRs than the respective reference categories. The highest HR within the BMI categories was observed for BMI ≥35.0 kg/m^2^ (HR 1.167, 95% CI 1.077–1.265), and the highest HR within the WC categories was observed for Category 6 (HR 1.195, 95% CI 1.157–1.235).

Pneumonia-related readmission estimates varied across BMI and WC categories (Table 3). Among male patients, several higher BMI categories had lower estimated HRs than the reference category. Among female patients, BMI categories from 23.0 to 34.9 kg/m^2^ also had lower HRs than the reference category, whereas the estimate for BMI ≥ 35.0 kg/m^2^ was higher but imprecise (HR 1.183, 95% CI 0.992–1.410).

Model-based mean costs of the first all-cause readmission varied across BMI and WC categories (Table 4). In the male stratum, the highest model-based mean cost across BMI categories was observed in the underweight category (USD 1771.4), whereas lower mean estimates were observed in several higher BMI categories. Across WC categories, the highest model-based mean cost in male patients was observed in Category 6 (USD 1500.8). Among female patients, the highest model-based mean cost across BMI categories was observed in the BMI 30.0–34.9 kg/m^2^ category (USD 1261.5), whereas the estimate for BMI ≥ 35.0 kg/m^2^ was USD 1162.7. Across WC categories, the highest model-based mean cost in female patients was observed in Category 6 (USD 1407.4).

Model-based mean pneumonia-related readmission costs also varied across BMI and WC categories (Table 5). Among male patients, costs declined from USD 1040.5 in the underweight group to USD 311.7 in the BMI ≥ 35.0 kg/m^2^ group. Female patients showed the highest pneumonia-related expenditure in the underweight category (USD 668.8) and the lowest in the BMI 30.0–34.9 kg/m^2^ category (USD 281.9). Across WC categories, the highest model-based mean pneumonia-related readmission cost was observed in Category 1 in both sex strata (USD 795.4 in males and USD 407.4 in females).

## 4. Discussion

This nationwide claims-based cohort study examined associations of BMI and WC with clinical and economic outcomes in analyses stratified by sex among South Korean adults with hospitalizations involving influenza or pneumonia. BMI and WC showed category-specific associations with readmission, while observed length of stay, index hospitalization costs, and model-based mean readmission costs varied descriptively across anthropometric categories and sex strata. BMI and WC showed different category-specific patterns when examined in separate models. Because they were not mutually adjusted, their independent or incremental contributions could not be determined. The male and female strata also differed in the distribution of index diagnoses and medical institution types, and these variables were not included in the regression models. Therefore, some apparent between-stratum differences may reflect differences in cohort composition, referral patterns, or underlying severity rather than effect modification by sex.

The inverse patterns observed across several higher BMI categories in male patients should be interpreted cautiously. Index hospitalization costs were summarized using unadjusted descriptive means; the readmission-cost estimates were not subjected to inferential between-category comparisons; and competing mortality was not modeled. Therefore, these findings do not establish a protective association of higher BMI with post-discharge outcomes. Because death precluded subsequent readmission and was not modeled using a competing-risk approach, the lower HRs observed in some higher BMI categories may have been influenced by differences in competing mortality. Although inverse associations between obesity and mortality have been reported in pneumonia populations, such findings may reflect selection, residual confounding, and competing mortality rather than a protective effect of adiposity. A meta-analysis by Nie et al. found that obesity was associated with increased pneumonia incidence but lower pneumonia-related mortality, suggesting that excess metabolic reserve or earlier clinical attention may contribute to better short-term outcomes in some settings [22]. However, this interpretation remains speculative and should be viewed cautiously. Lower BMI in hospitalized patients may reflect frailty, chronic illness, sarcopenia, or illness-related weight loss, all of which may be associated with worse outcomes independent of adiposity itself.

In the female stratum, the observed WC pattern was more consistent for all-cause readmission than for pneumonia-related readmission. One possible explanation is that WC reflects abdominal adiposity, which has been associated with chronic inflammation, altered immune responses, and impaired respiratory mechanics, while sex-related differences in visceral and subcutaneous fat distribution may also contribute to the observed pattern [4,10,12,23]. However, this interpretation remains speculative because WC was not mutually adjusted for BMI, no formal sex-by-WC interaction test was performed, and detailed physiological, behavioral, and disease-severity data were unavailable. Prior studies have linked visceral or central obesity to adverse outcomes in infection-related and postoperative settings [24,25,26,27,28]. In the present study, higher WC categories were associated with greater all-cause readmission burden in the female stratum, and model-based mean costs of the first all-cause readmission were higher in the upper WC categories. These category-specific observational associations do not establish clinical or predictive utility; mutually adjusted models and validation in independent cohorts are needed to determine whether WC provides information beyond BMI.

The distinction between all-cause and pneumonia-related readmission may help explain the differing category-specific patterns across outcomes. Previous studies have shown that readmissions after pneumonia are heterogeneous and may involve recurrent pneumonia as well as cardiovascular, respiratory, renal, and other comorbidity-related conditions [29,30,31]. Accordingly, all-cause readmission may capture a broader spectrum of post-discharge morbidity and healthcare use, whereas pneumonia-related readmission represents a more specific and less frequent claims-defined outcome. In the present cohort, 56.8% of patients experienced all-cause readmission, whereas 15.3% experienced pneumonia-related readmission. The WC pattern observed for all-cause readmission in the female stratum, together with the less consistent pneumonia-related pattern, may therefore reflect an association with overall post-discharge morbidity rather than recurrent pneumonia alone. This interpretation remains tentative because the diagnoses underlying all-cause readmissions were not classified, and planned admissions, disease severity, and competing mortality could not be fully addressed.

BMI and WC showed different category-specific patterns when examined separately. However, the present study did not assess discrimination, calibration, reclassification, or the incremental predictive value of WC beyond BMI. The observed associations, therefore, warrant further epidemiological and prognostic investigation but do not establish clinical utility for risk stratification or post-discharge management [14,32].

The economic findings should be interpreted in light of the outcome definition. Readmission-related costs reflected medical expenditures derived from claims data for the first qualifying readmission within two years after discharge, rather than cumulative expenditures across all subsequent hospitalizations. Accordingly, the reported estimates characterize the cost of the first qualifying post-discharge hospitalization and may underestimate the overall post-discharge economic burden among patients with multiple readmissions. Future studies using recurrent-event and cumulative-cost approaches are warranted to provide a more comprehensive evaluation of the economic burden across BMI and WC categories.

This study has several strengths. It used a large nationwide cohort with linkage between administrative claims and pre-admission health-screening data, enabling simultaneous assessment of BMI and WC in relation to clinical and economic outcomes. Presenting results separately by sex enabled descriptive comparison of category-specific patterns, although it did not establish effect modification. At the same time, several limitations should be considered. First, residual confounding is possible because claims data lacked information on vaccination history, smoking status, infection severity, oxygen requirements, radiologic findings, intensive care unit admission, frailty, recent weight loss, and functional status. Second, BMI and WC were obtained from health examinations conducted up to two years before hospitalization and may not fully reflect body composition at the time of infection, which may have introduced exposure misclassification. Changes in body weight, muscle mass, abdominal adiposity, or fluid status during the interval may have resulted in discordance between the recorded BMI or WC measurements and adiposity at admission. Third, the inclusion of primary and secondary diagnosis codes may have increased case capture but may also have included patients in whom influenza or pneumonia was not the primary reason for admission; a sensitivity analysis restricted to primary diagnosis codes would strengthen future analyses. Fourth, during the two-year follow-up, 12,996 patients (7.4%) died, and mortality differed substantially across BMI categories. Death was treated as a censoring event in conventional Cox models but was not analyzed using a competing-risk framework; consequently, differential mortality may have biased the readmission estimates. Although BMI- and WC-stratified survival curves showed no marked crossing, formal residual-based diagnostics of the proportional hazards assumption were not performed. Modest nonproportionality therefore cannot be excluded, and the reported HRs should be interpreted as average associations over the two-year follow-up period rather than as associations that remained constant over time. Fifth, because the analysis was limited to individuals with available health-screening data, selection bias is possible. The cohort was restricted to participants in the NHIS screening program. Screening participants may differ from non-participants in socioeconomic and employment status, preventive-care use, healthcare engagement, health literacy, health behaviors, functional status, and comorbidity burden. These differences may limit generalizability and may also have influenced the observed associations. Finally, findings may not be directly generalizable to non-Korean or non-Asian populations, given differences in adiposity distribution, obesity-related risk profiles, and BMI classification thresholds.

Future research should investigate mechanisms linking central obesity to adverse post-discharge outcomes after respiratory infection, including pathways related to inflammation, immune dysregulation, pulmonary function, and healthcare utilization. Future studies should use more recent or temporally well-defined pre-index measurements, competing-risk methods, mutually adjusted BMI and WC models, formal interaction and nonlinear analyses, more specific diagnosis definitions, and validated prediction models.

## 5. Conclusions

In this nationwide claims-based cohort of adults in Korea with hospitalizations involving influenza or pneumonia, BMI and WC showed category-specific associations with readmission, while model-based mean readmission costs varied across anthropometric categories, outcomes, and sex strata. Higher WC categories were associated with greater all-cause readmission burden in the female stratum, whereas BMI showed different patterns across the male and female strata. These findings support further epidemiological evaluation of BMI and WC in relation to post-discharge healthcare outcomes. Because BMI and WC were analyzed separately and formal interaction, competing-risk, and prediction analyses were not performed, the findings do not establish sex-based effect modification or the independent or incremental value of WC beyond BMI. Further studies using more recent pre-admission measurements, mutually adjusted models, competing-risk methods, and prospective validation are needed to clarify the reproducibility and prognostic relevance of these associations.

## Figures and Tables

**Table 1:** Baseline characteristics of adults hospitalized for influenza or pneumonia.

	Male	Female	Total	*p*-Value
	N (%)	N (%)	N (%)	
**N**	80,257	95,517	175,774	
**Age (years)**				
**(Mean ± SD)**	63.3 ± 16.2	61.9 ± 16.6	62.6 ± 16.4	<0.001
**20–49**	16,991 (21.2)	21,249 (22.2)	38,240 (21.8)	<0.001
**50–64**	20,901 (26.0)	29,696 (31.1)	50,597 (28.8)	
**≥65**	42,365 (52.8)	44,572 (46.7)	86,937 (49.5)	
**Body mass index (kg/m^2^)**				
**(Mean ± SD)**	23.7 ± 3.6	23.9 ± 3.9	23.8 ± 3.8	<0.001
**<18.5**	5281 (6.6)	5754 (6.0)	11,035 (6.3)	<0.001
**18.5–22.9**	28,572 (35.6)	35,885 (37.6)	64,457 (36.7)	
**23.0–24.9**	18,507 (23.1)	20,003 (20.9)	38,510 (21.9)	
**25.0–29.9**	24,245 (30.2)	27,302 (28.6)	51,547 (29.3)	
**30.0–34.9**	3228 (4.0)	5533 (5.8)	8761 (5.0)	
**≥35.0**	424 (0.5)	1040 (1.1)	1464 (0.8)	
**Waist circumference (cm)**				
**(Mean ± SD)**	84.7 ± 9.4	79.8 ± 10.1	82.0 ± 10.1	<0.001
**Category 1**	22,640 (28.2)	30,321 (31.7)	52,961 (30.1)	<0.001
**Category 2**	17,461 (21.8)	17,863 (18.7)	35,324 (20.1)	
**Category 3**	16,863 (21.0)	18,418 (19.3)	35,281 (20.1)	
**Category 4**	12,330 (15.4)	13,051 (13.7)	25,381 (14.4)	
**Category 5**	6409 (8.0)	8404 (8.8)	14,813 (8.4)	
**Category 6**	4554 (5.7)	7460 (7.8)	12,014 (6.8)	
**Diagnosis**				
**Influenza**	22,281 (27.8)	38,269 (40.1)	60,550 (34.4)	<0.001
**Pneumonia**	57,976 (72.2)	57,248 (59.9)	115,224 (65.6)	
**Charlson Comorbidity Index**				
**(Mean ± SD)**	2.0 ± 1.9	1.8 ± 1.7	1.9 ± 1.8	<0.001
**0**	20,502 (25.5)	23,618 (24.7)	44,120 (25.1)	<0.001
**1–2**	34,143 (42.5)	44,687 (46.8)	78,830 (44.8)	
**≥3**	25,612 (31.9)	27,212 (28.5)	52,824 (30.1)	
**Income level**				
**Low**	18,009 (22.4)	25,108 (26.3)	43,117 (24.5)	<0.001
**Middle–low**	13,685 (17.1)	17,288 (18.1)	30,973 (17.6)	
**Middle–high**	19,629 (24.5)	21,439 (22.4)	41,068 (23.4)	
**High**	27,491 (34.3)	29,919 (31.3)	57,410 (32.7)	
**Type of residential area**				
**Metropolitan**	55,059 (68.6)	68,757 (72.0)	123,816 (70.4)	<0.001
**Non-metropolitan**	25,198 (31.4)	26,760 (28.0)	51,958 (29.6)	
**Type of medical institution**				
**Tertiary hospital**	10,842 (13.5)	7901 (8.3)	18,743 (10.7)	<0.001
**General hospital**	42,081 (52.4)	45,424 (47.6)	87,505 (49.8)	
**Hospital**	23,871 (29.7)	35,701 (37.4)	59,572 (33.9)	
**Clinic**	3463 (4.3)	6491 (6.8)	9954 (5.7)	

SD, standard deviation.

**Table 2:** All-cause readmission within two years by obesity level among adults hospitalized for influenza or pneumonia.

	Total N	Follow-Up Years	Cases	Incidence (per 1000 Person-Years)	Hazard Ratio (95%CI)
**Male**					
**Body mass index (kg/** **m^2^** **)**					
**<18.5**	5281	5368	3407	634.7	1.091 (1.052–1.132)
**18.5–22.9**	28,572	32,369	16,686	515.5	Ref.
**23.0–24.9**	18,507	22,229	10,276	462.3	0.958 (0.935–0.982)
**25.0–29.9**	24,245	30,250	13,041	431.1	0.951 (0.930–0.974)
**30.0–34.9**	3228	4173	1665	399.0	1.020 (0.969–1.073)
**≥35.0**	424	565	207	366.4	1.137 (0.991–1.305)
**Waist circumference (cm)**					
**Category 1**	22,640	26,516	12,659	477.4	Ref.
**Category 2**	17,461	21,052	9596	455.8	0.970 (0.944–0.996)
**Category 3**	16,863	20,181	9444	468.0	0.974 (0.948–1.000)
**Category 4**	12,330	14,615	7067	483.5	0.982 (0.954–1.011)
**Category 5**	6409	7395	3813	515.6	1.029 (0.992–1.067)
**Category 6**	4554	5194	2703	520.4	1.072 (1.028–1.118)
**Female**					
**Body mass index (kg/m^2^)**					
**<18.5**	5754	6781	3228	476.0	1.039 (1.001–1.078)
**18.5–22.9**	35,885	45,406	19,299	425.0	Ref.
**23.0–24.9**	20,003	24,185	11,603	479.8	1.034 (1.011–1.058)
**25.0–29.9**	27,302	32,055	16,487	514.3	1.072 (1.050–1.094)
**30.0–34.9**	5533	6421	3382	526.7	1.124 (1.084–1.166)
**≥35.0**	1040	1251	617	493.4	1.167 (1.077–1.265)
**Waist circumference (cm)**					
**Category 1**	30,321	40,104	15,260	380.5	Ref.
**Category 2**	17,863	22,176	10,031	452.3	1.040 (1.014–1.067)
**Category 3**	18,418	21,703	10,974	505.6	1.079 (1.053–1.107)
**Category 4**	13,051	14,763	8134	551.0	1.130 (1.100–1.162)
**Category 5**	8404	9318	5356	574.8	1.140 (1.104–1.177)
**Category 6**	7460	8034	4861	605.0	1.195 (1.157–1.235)

Hazard ratios were calculated using Cox proportional hazards models, adjusting for age, Charlson Comorbidity Index score, income level, and residential area type. CI, confidence interval; Ref, reference.

**Table 3:** Pneumonia-related readmission within two years by obesity level among adults hospitalized for influenza or pneumonia.

	Total N	Follow-Up Years	Cases	Incidence (per 1000 Person-Years)	Hazard Ratio (95%CI)
**Male**					
**Body mass index (kg/m^2^)**					
**<18.5**	5281	7619	1729	226.9	1.414 (1.341–1.492)
**18.5–22.9**	28,572	46,553	6311	135.6	Ref.
**23.0–24.9**	18,507	31,898	3073	96.3	0.797 (0.763–0.832)
**25.0–29.9**	24,245	43,231	3225	74.6	0.699 (0.669–0.729)
**30.0–34.9**	3228	5915	345	58.3	0.719 (0.645–0.802)
**≥35.0**	424	800	34	42.5	0.777 (0.554–1.089)
**Waist circumference (cm)**					
**Category 1**	22,640	36,828	4978	135.2	Ref.
**Category 2**	17,461	29,871	3062	102.5	0.795 (0.760–0.832)
**Category 3**	16,863	29,034	2823	97.2	0.738 (0.705–0.773)
**Category 4**	12,330	21,379	2006	93.8	0.694 (0.659–0.731)
**Category 5**	6409	11,003	1091	99.2	0.723 (0.677–0.772)
**Category 6**	4554	7902	757	95.8	0.737 (0.682–0.796)
**Female**					
**Body mass index (kg/m^2^)**					
**<18.5**	5754	9404	1264	134.4	1.587 (1.491–1.690)
**18.5–22.9**	35,885	64,741	4525	69.9	Ref.
**23.0–24.9**	20,003	36,387	2370	65.1	0.852 (0.810–0.895)
**25.0–29.9**	27,302	49,852	3210	64.4	0.814 (0.778–0.852)
**30.0–34.9**	5533	10,174	604	59.4	0.808 (0.742–0.880)
**≥35.0**	1040	1891	129	68.2	1.183 (0.992–1.410)
**Waist circumference (cm)**					
**Category 1**	30,321	55,079	3610	65.5	Ref.
**Category 2**	17,863	32,416	2126	65.6	0.807 (0.765–0.852)
**Category 3**	18,418	33,148	2405	72.6	0.793 (0.753–0.836)
**Category 4**	13,051	23,462	1732	73.8	0.762 (0.720–0.808)
**Category 5**	8404	15,032	1157	77.0	0.759 (0.710–0.811)
**Category 6**	7460	13,311	1072	80.5	0.812 (0.758–0.870)

Hazard ratios were calculated using Cox proportional hazards models, adjusting for age, Charlson Comorbidity Index score, income level, and residential area type. CI, confidence interval; Ref, reference.

**Table 4:** All-cause readmission costs within two years estimated using a two-part model (unit: 2018 USD).

	Mean	Resampling SD	95% CI of Resampled Mean	Cost Ratio
**Male**				
**Body mass index (kg/m^2^)**				
**<18.5**	1771.4	5.9	1771.1–1771.8	1.177
**18.5–22.9**	1505.3	3.5	1505.0–1505.5	Ref.
**23.0–24.9**	1436.5	4.9	1436.2–1436.8	0.954
**25.0–29.9**	1328.1	4.3	1327.9–1328.4	0.882
**30.0–34.9**	1221.6	13.3	1220.8–1222.5	0.812
**≥35.0**	1158.0	35.5	1155.8–1160.2	0.769
**Waist circumference (cm)**				
**Category 1**	1471.7	4.2	1471.5–1472.0	Ref.
**Category 2**	1391.8	5.0	1391.5–1392.1	0.946
**Category 3**	1428.9	5.0	1428.6–1429.2	0.971
**Category 4**	1461.8	5.7	1461.4–1462.2	0.993
**Category 5**	1434.9	7.2	1434.5–1435.4	0.975
**Category 6**	1500.8	9.4	1500.2–1501.3	1.020
**Female**				
**Body mass index (kg/m^2^)**				
**<18.5**	1252.1	6.3	1251.7–1252.5	1.120
**18.5–22.9**	1118.0	3.3	1117.8–1118.2	Ref.
**23.0–24.9**	1148.6	3.7	1148.3–1148.8	1.027
**25.0–29.9**	1255.7	3.1	1255.5–1255.9	1.123
**30.0–34.9**	1261.5	7.7	1261.1–1262.0	1.128
**≥35.0**	1162.7	17.1	1161.6–1163.8	1.040
**Waist circumference (cm)**				
**Category 1**	979.2	3.3	979.0–979.4	Ref.
**Category 2**	1162.5	4.3	1162.2–1162.8	1.187
**Category 3**	1256.2	3.9	1255.9–1256.4	1.283
**Category 4**	1295.4	4.3	1295.1–1295.6	1.323
**Category 5**	1309.7	4.5	1309.5–1310.0	1.338
**Category 6**	1407.4	5.5	1407.0–1407.7	1.437

Readmission costs were estimated using a two-part model, and mean values were calculated based on 1000 bootstrap resamples. The SD is the standard deviation of 1000 resampled mean estimates, and the 95% CI is the confidence interval for the mean of those estimates. These values reflect resampling stability rather than full model-based sampling uncertainty. Costs are presented as 2018 USD.

**Table 5:** Pneumonia-related readmission costs within two years estimated using a two-part model (unit: 2018 USD).

	Mean	Resampling SD	95% CI of Resampled Mean	Cost Ratio
**Male**				
**Body mass index (kg/m^2^)**				
**<18.5**	1040.5	6.6	1040.1–1040.9	1.439
**18.5–22.9**	723.3	3.3	723.1–723.5	Ref.
**23.0–24.9**	627.6	4.5	627.4–627.9	0.868
**25.0–29.9**	512.1	4.2	511.8–512.3	0.708
**30.0–34.9**	453.8	14.3	452.9–454.7	0.627
**≥35.0**	311.7	27.3	310.0–313.3	0.431
**Waist circumference (cm)**				
**Category 1**	795.4	4.6	795.1–795.7	Ref.
**Category 2**	620.5	4.8	620.2–620.8	0.780
**Category 3**	638.5	4.8	638.2–638.8	0.803
**Category 4**	575.1	5.2	574.8–575.4	0.723
**Category 5**	587.6	6.3	587.2–588.0	0.739
**Category 6**	602.5	9.4	601.9–603.0	0.757
**Female**				
**Body mass index (kg/m^2^)**				
**<18.5**	668.8	5.7	668.5–669.2	1.624
**18.5–22.9**	411.9	3.0	411.7–412.1	Ref.
**23.0–24.9**	326.1	3.1	325.9–326.3	0.792
**25.0–29.9**	324.7	2.8	324.6–324.9	0.788
**30.0–34.9**	281.9	6.4	281.5–282.3	0.684
**≥35.0**	291.8	13.9	290.9–292.6	0.708
**Waist circumference (cm)**				
**Category 1**	407.4	3.7	407.2–407.6	Ref.
**Category 2**	379.1	4.3	378.8–379.3	0.930
**Category 3**	363.5	3.1	363.3–363.7	0.892
**Category 4**	354.2	3.5	354.0–354.5	0.870
**Category 5**	361.7	4.1	361.4–362.0	0.888
**Category 6**	384.3	5.2	384.0–384.6	0.943

Readmission costs were estimated using a two-part model, and mean values were calculated based on 1000 bootstrap resamples. The SD is the standard deviation of 1000 resampled mean estimates, and the 95% CI is the confidence interval for the mean of those estimates. These values reflect resampling stability rather than full model-based sampling uncertainty. Costs are presented as 2018 USD.

## Data Availability

The data that support the findings of this study are not publicly available due to legal, privacy, and data-use restrictions imposed by the National Health Insurance Service of Korea. Researchers may apply for access to the NHIS Customized Research Database through the official review and approval process.

## Abbreviations

The following abbreviations are used in this manuscript:

ATC	Anatomical Therapeutic Chemical
BMI	Body Mass Index
CCI	Charlson Comorbidity Index
CI	Confidence Interval
HR	Hazard Ratio
ICD-10	International Classification of Diseases, 10th Revision
KSSO	Korean Society for the Study of Obesity
KRW	Korean Won
NHIS	National Health Insurance Service
SD	Standard Deviation
USD	US Dollar
WC	Waist Circumference
